# The Effects of Cold and Lower Body Negative Pressure on Cardiovascular Homeostasis

**DOI:** 10.1155/2015/728145

**Published:** 2015-03-19

**Authors:** David J. Kean, Corey A. Peacock, Gabriel J. Sanders, John McDaniel, Lisa A. C. Colvin, Ellen L. Glickman

**Affiliations:** ^1^Exercise and Environmental Physiology Laboratory, Kent State University, Kent, OH 44242, USA; ^2^Department of Kinesiology, University of Louisiana at Monroe, 700 University Avenue, Monroe, LA 71209, USA; ^3^Farquhar College of Arts and Sciences, Nova Southeastern University, Fort Lauderdale-Davie, FL 33314, USA; ^4^Department of Kinesiology and Health, Northern Kentucky University, Highland Heights, KY 41099, USA

## Abstract

*Purpose*. The purpose of this study is to determine how cold exposure and lower body negative pressure effected cardiovascular variables. *Methods*. Eleven males (20.3 years ± 2.7) underwent two 20-minute exposures to LBNP. During the 2 trials, the subjects were exposed to cold air (10°C) (COLD) and to ambient temperature (23°C) (AMB). The trials consisted of a 100-minute pre-LBNP period followed by a 20-minute exposure to LBNP and then a 15-minute recovery period. Cardiovascular variables were recorded every 30 minutes using bioimpedance. *Results*. When LBNP was applied during the AMB trials, stroke volume immediately decreased. During the COLD trial, there was a five-minute delay before the decrease in stroke volume. Heart rate increased immediately after LBNP initiation during the AMB trials but there was a delay in the increase during the COLD trials. That same pattern was followed with mean arterial blood pressures. Cerebral oxygenation was significantly lower throughout the COLD trial as compared to the AMB trials. Six subjects reported symptoms of syncope or presyncope during the AMB trials but there were no reports of symptoms during the COLD trials. *Conclusion*. From analysis of this data, cold improved the subject's tolerance to LBNP.

## 1. Introduction

Hemorrhage is a leading cause of death in both civilian and battlefield trauma. Survival rate increases when victims requiring immediate intervention are correctly identified in a mass-casualty situation, but methods of prioritizing casualties based on current triage algorithms are severely limited [1]. Controlled study of acute hemorrhage in humans is not possible; however, it is possible to safely simulate hemorrhage by applying negative pressure to the lower extremities. Specifically, lower body negative pressure (LBNP) sequesters blood from the thorax into dependent regions of the pelvis and legs, effectively decreasing central blood volume in a similar fashion to hemorrhage [1].

It is well established that a thermal stressor elicits physiological responses that alter central blood volume. As environmental temperature decreases or length of exposure increases, the body attempts to maintain thermoregulatory homeostasis via multiple mechanisms [2]. Specifically, the peripheral vasculature will constrict, shunting warmer blood to the core to maintain core temperature, the onset of metabolic heat production, and shivering thermogenesis [2]. When environmental temperature increases, the body will attempt to maintain thermoregulation through peripheral vasodilation and sweating [2].

Optimal management of hemorrhage and controlling the effects of a reduction in central blood volume require recognition and integration of multiple complex physiological responses. The current therapy, which is warming the patient, used in the majority of cases has shown the potential to cause other issues and has been challenged [1, 3].

Numerous studies have looked at the effects that cold has on tolerance of LBNP. The majority have used a reduction in skin temperature and controlled the core temperature to prevent a decrease and then subsequent shivering thermogenesis [4–7].

The purpose of this investigation was to determine the effects that decreasing the body's core temperature has on tolerance of exposure to LBNP and the subsequent reduction in central blood volume and whether that response is different from previous studies.

## 2. Materials and Methods

### 2.1. Subjects

Eleven Caucasian male subjects between the ages of 18 and 25 years volunteered for this investigation. Only male subjects were used to limit thermoregulatory variables. Participant characteristics are listed in Table 1. All subjects were free of disease and were not taking any medication that will influence cardiovascular or fluid regulation. Health status was determined via a self-reported health history questionnaire. Each subject was provided with and read and signed an informed consent in accordance with the guidelines set forth by the Kent State University Human Subjects Review Board.

### 2.2. Design

Participants underwent an exposure to LBNP in a cold experimental trial in one visit (COLD) and an exposure to LBNP in an ambient experimental trial (AMB) in another visit. Both trials were staged in the Environmental Chamber (Neslab, Napa, California), Figure 7, in the Kent State Environmental Laboratory. The order was determined in a counterbalanced manner. Participants refrained from exercise and from consuming any alcohol or caffeine 24 hours prior to the start of either trial. The two trials (COLD and AMB) were separated by a minimum of 48 hours so that the effects of prior cold exposure, which was confirmed verbally, would not alter tolerance of temperature or of LBNP with the subsequent trial which was verified verbally prior to the start of the second trial.

### 2.3. Instrumentation

Prior to the start of the experimental trial, participants were instructed to insert a rectal thermometer (ER 400-12, O.E. Meyer Co., Sandusky, OH) 13 cm past the anal sphincter for continuous measurement of core temperature. Skin thermistors (Model number 409 B, Yellow Springs Instruments, Inc., Yellow Springs, OH) were applied to the individual's body and held in place by waterproof tape (Hy-tape International, Patterson, NY). The thermistors were applied to the right forearm, triceps, chest, thigh, and calf [8]. Skin thermistors were applied and the participants were fitted with a Polar heart rate monitor (Accurex Plus, Polar Electro, Inc., Woodbury, NY) which was used to monitor heart rate. Bioimpedance sensors (Cardiodynamics BioZ, San Diego, CA) [9] were placed on both carotid arteries and the axillary regions so that mean arterial blood pressure and stroke volume were monitored using impedance cardiography. The INVOS system was used for real-time monitoring of changes in regional brain oxygen saturation (rSO_2_) of blood flow in the frontal lobe of the brain (Somanetics Corp., Troy, MI) [10]. Subjects were fitted with one sensor on their forehead prior to the start of the experimental trial and it remained there until completion of the trial.

### 2.4. Protocol

Subjects were dressed in athletic shorts only for both trials. Subjects positioned themselves in the LBNP box, which is pictured in Figure 8, up to the level of the anterior superior iliac spine. The subject then lied quietly on a table (Hausmann Industries, Northvale, NJ) for the duration of the trial. LBNP was applied at a level of −40 mmHg for both trials. The LBNP box was placed in the chamber prior to cooling and, for both trials, the top of the box was removed for the initial exposure period. This insured that there was not any increase in core temperature due to being sheltered from the environmental stress. The COLD experimental trial consisted of two components: an initial acute cold exposure (ACE) in an environmentally controlled chamber (Neslab, Napa, CA) followed by the LBNP application period. The acute cold exposure was to last for 100 minutes unless the subject's core temperature dropped 1°C at which time the LBNP application was initiated. LBNP application was terminated after 20 minutes or if the subject began experiencing nausea or lost consciousness. The environmental chamber was controlled within ±3°C of 10°C. There was a 15 min recovery period after the trial. The AMB experimental trial took place in the chamber with the temperature controlled within ±5°C of 23°C. LBNP was applied 100 minutes after initiation of the trial and terminated after 20 minutes. There was a 15-minute-recovery period after the AMB trial.

### 2.5. Data Analysis

Means and standard deviations were calculated for subject characteristics (age, height, mass, body surface area, surface area-to-mass ratio, body fat percentages, and VO_2_). The study included two treatments, LBNP in ambient temperature and LBNP in cold. A group of male college aged students (*N* = 11) were subjects. All data were analyzed with SPSS software version 17 (Chicago, Ill). Two separate two-way repeated measures analysis of variance (ANOVA) by 10 time-points (0, 5, 20, 35, 50, 65, 80, 95, 110, and 125 minutes) with two conditions (AMB and COLD), were utilized to examine differences in *T*_re_ and *T*_sk_.

Repeated measures analysis of variance was used to determine if there was a difference in dependent variables across time and condition (COLD and AMB). Specifically, a 2 × 8 repeated measures ANOVA was used to analyze mean arterial blood pressure, heart rate, stroke volume, and regional oxygen saturation. For all comparisons, alpha was set at 0.05.

## 3. Results

### 3.1. Rectal and Skin Temperature

**Figure 1:**
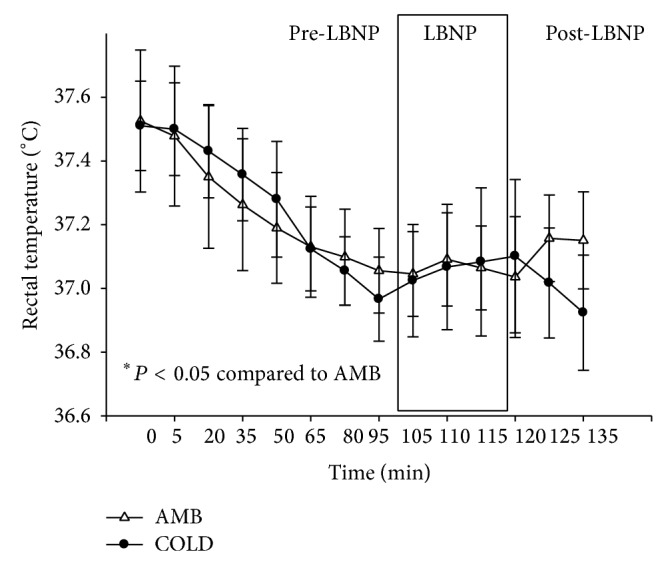
Changes in rectal temperature (°C) (M ± 95% CI) during pre-LBNP, LBNP, and post-LBNP periods.

**Figure 2:**
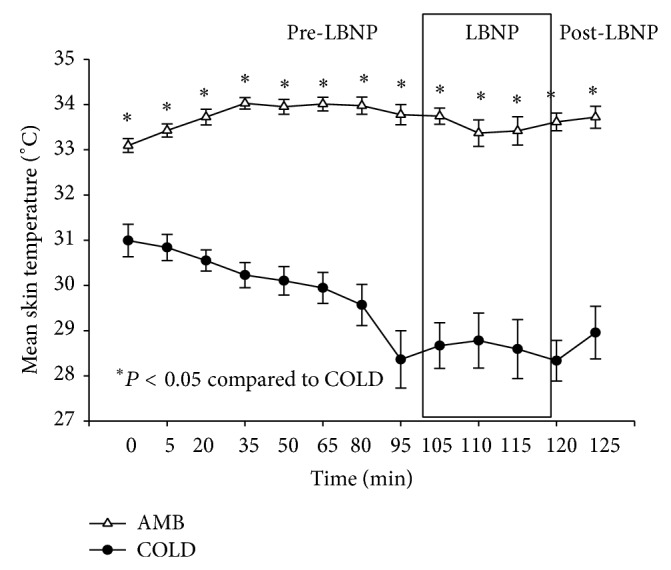
Changes in mean skin temperature (°C) (M ± 95% CI) during pre-LBNP, LBNP, and post-LBNP periods.

Repeated measures ANOVA demonstrated that there was a significant condition by time interaction (*P* < 0.05) for both rectal and skin temperature (Figures 1 and 2). Rectal temperature decreased with time but was not different between conditions. Subjects core temperatures did drop more than 1°C during the COLD trials so all subjects were initially exposed to cold for 100 minutes. However, skin temperature decreased during COLD trial but did not change across time during the AMB trial. Skin temperature was significantly less at each time point for the COLD trial as compared to the AMB trial.

A Shapiro-Wilk's test (*P* > 0.05) [11] showed that the temperature readings were approximately normally distributed with the exception of the readings at one hundred thirty-five minutes of the AMB trials. Partial eta squared was 0.334.

### 3.2. Mean Arterial Blood Pressure (MAP)

**Figure 3:**
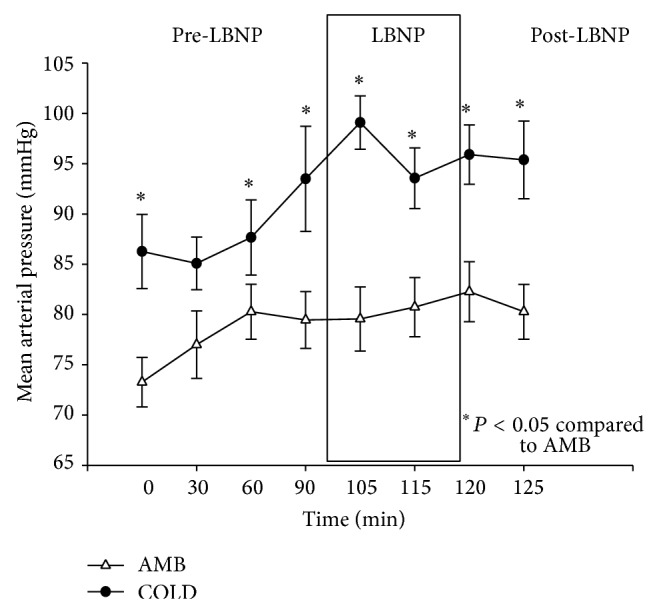
Changes in mean arterial blood pressure (mmHg) (M ± 95% CI) during the pre-LBNP, LBNP, and post-LBNP periods.

MAP increased during the pre-LBNP period for both the COLD and AMB trials. The data for MAP is presented in Figure 3. Repeated measures ANOVA demonstrated a significant condition by time interaction (*P* < 0.05). The average change in pressure was 6.18 mmHg for the AMB trials and 7.91 mmHg for the COLD trials. MAP leveled off after the 60-minute measurement during the AMB trials. There was an average increase of 1.27 mmHg during the LBNP application for the AMB trials and a decrease of 0.45 mmHg during the post-LBNP period.

During the COLD trials, there was an initial increase in MAP (on average, the increase was 5 mmHg) 5 minutes after application of LBNP followed by an average decrease of 6 mmHg before the cessation of LBNP. This was likely due to a further shift in fluids to the core with LBNP. The overall average change in MAP during LBNP application was −1 mmHg on average.

A Shapiro-Wilk's test (*P* > 0.05) [9] showed that mean arterial pressures were approximately normally distributed with the exception of the reading at the sixty-minute mark of the AMB trials. Partial eta squared was 0.290.

### 3.3. Heart Rate

**Figure 4:**
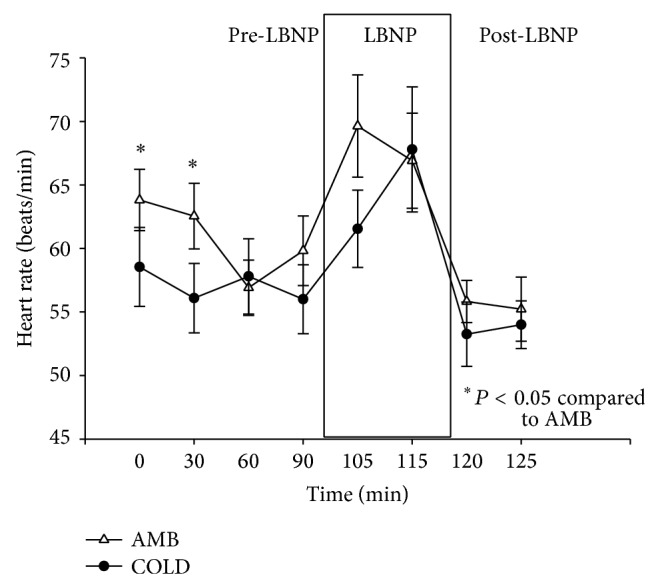
Changes in heart rate (beats/min) (M ± 95% CI) during the pre-LBNP, LBNP, and post-LBNP periods.

Heart rate decreased by an average of 4 bpm on average during the pre-LBNP period of the AMB trials and by an average of 1 bpm on average during the COLD trials. The data for heart rate is presented in Figure 4. Heart rate dropped at the 60-minute measurement during the AMB trial before a sharp increase prior to the application of LBNP. Heart rate stayed steady during the pre-LBNP period of the COLD trials. Repeated measures ANOVA demonstrated that there was not a significant condition by time interaction (*P* > 0.05).

A Shapiro-Wilk's test (*P* > 0.05) [9] showed that heart rates were approximately normally distributed. Partial eta squared was 0.148.

### 3.4. Stroke Volume

**Figure 5:**
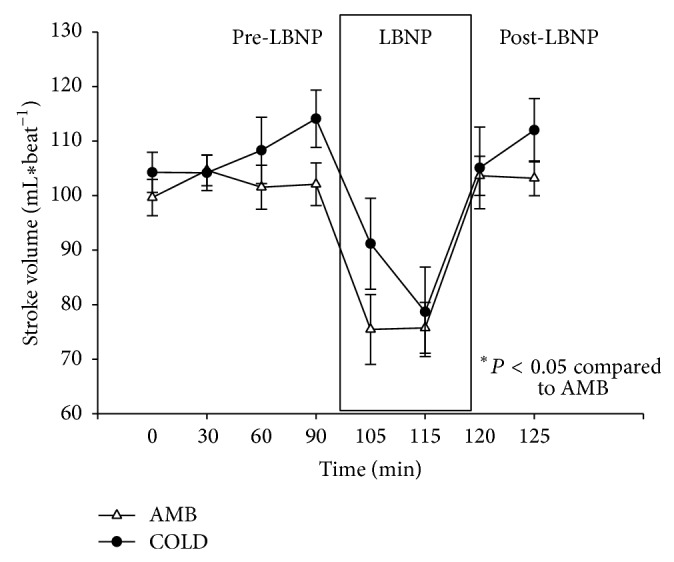
Changes in stroke volume (mL × beat^−1^) (M ± 95% CI) during the pre-LBNP, LBNP, and post-LBNP periods.

During the pre-LBNP period, there was an average increase of 2.45 mL/beat during the AMB trials. The data for stroke volume is presented in Figure 5. There was an average increase of 5 mL/beat during the pre-LBNP of the COLD trials. Repeated measures ANOVA demonstrated that there was not a significant condition by time interaction (*P* > 0.05).

A Shapiro-Wilk's test (*P* > 0.05) [9] showed that stroke volumes were approximately normally distributed with the exception of values taken at the thirty-minute mark and the one hundred fifteen-minute mark during COLD trials. Partial eta squared was 0.196.

### 3.5. Cerebral Oxygenation

**Figure 6:**
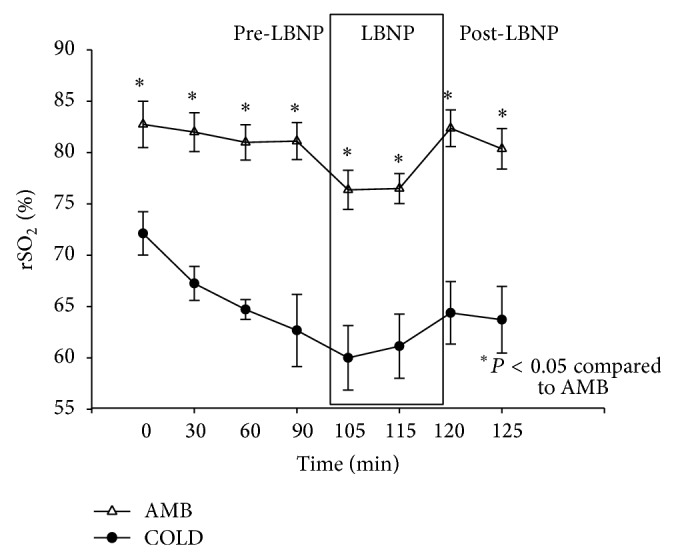
Changes in cerebral oxygenation (%) (M ± 95% CI) during the pre-LBNP, LBNP, and post-LBNP periods.

**Figure 7:**
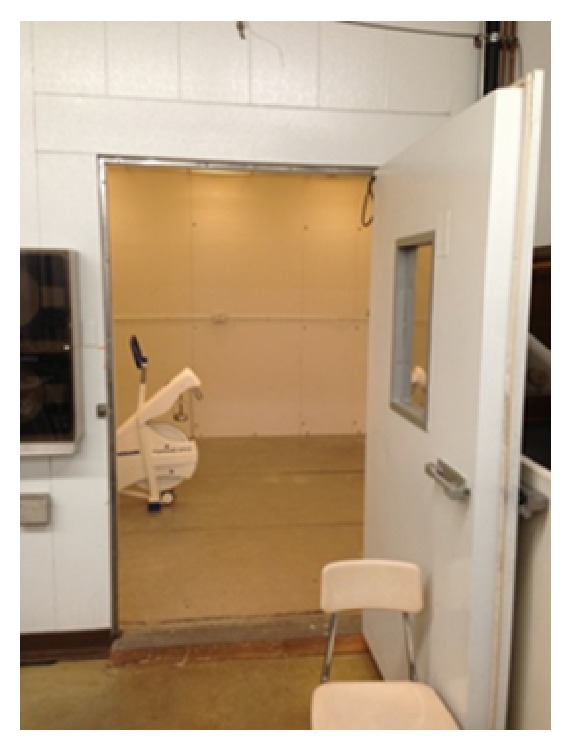
The exterior of the environmental chamber at the Kent State Environmental Laboratory.

**Figure 8:**
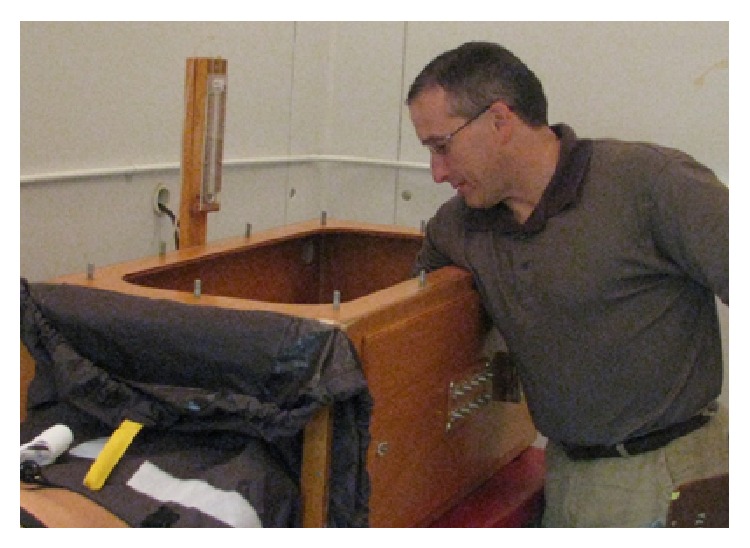
The LBNP box.

During the pre-LBNP period, there was an average decrease of 1.62% during the AMB trials and an average decrease of 9% during the COLD trials. Application of LBNP caused an average decrease of 4.63% during the AMB trials. All of the AMB readings were significantly higher than the COLD readings. The data for cerebral oxygenation is presented in Figure 6.

During the COLD trials, there was an average decrease of 1%. An average increase of 3.88% was observed during the post-LBNP period of the AMB trials. There was an average increase of 2.57% during the post-LBNP period. There was a significant condition by time interaction (*P* < 0.05).

A Shapiro-Wilk's test (*P* > 0.05) [9] showed that stroke volumes were approximately normally distributed with the exception of the readings at ninety minutes and one hundred twenty-five minutes during the COLD trials. Partial eta squared was 0.333.

## 4. Discussion

The present investigation was conducted to evaluate the effects of cold exposure and lower body negative pressure (LBNP) on cardiovascular and thermoregulatory homeostasis. The subjects in this study were exposed to cold for 100 minutes prior to exposure to LBNP in an effort to decrease core temperature and evaluate the effects on a simulated hemorrhage. When an individual is exposed to the cold, both central and peripheral mechanisms are utilized so that core temperature may be maintained. In an effort to retain core temperature, shivering thermogenesis, in part, increases metabolic heat production while peripheral vasoconstriction minimizes heat loss [2].

This may be an advantage in treatment of hemorrhage. The resultant reduction in central blood volume from hemorrhage elicits a left and upward shift of the Frank-Starling curve, which leaves the cardiovascular system at risk for collapse with any further reduction in volume [12]. The mechanisms that the body uses in maintenance of core temperature during cold exposure should attenuate this.

Hemorrhage leads typically to a reduction in central blood volume. The response of the body is to maintain perfusion of tissues. In order to do that, blood pressure must be maintained. The data in this investigation indicates that, during a simulated hemorrhage (LBNP) in ambient temperature, there was a decrease in stroke volume, rSO_2_, which is indicative of a fluid shift away from the core. The reduction in central blood volume and blood pressure causes compromised perfusion of tissues [4]. Cerebral blood flow velocity was measured in a previous study by Wilson et al. [13]. They used a head-up tilt to create the blood pooling in the lower extremities. They used a tube lined suit for skin surface cooling and found that cerebral blood flow velocity was unchanged when the skin surface was cooled. They also reported that the subjects did not demonstrate symptoms of syncope with the cold exposure. Anecdotally, there were 6 subjects who reported symptoms of syncope during the AMB trials, but there were no episodes of syncopal symptoms during the COLD trials.

A previous study by László et al. [14] suggested that there was no significant increase in MAP with LBNP at −35 mmHg. When the pressure increased to −55 and −65 mmHg, there was an increase in MAP, so they concluded that there was a dose-response relationship with LBNP and the cardiovascular system. The results of the present investigation agree with studies done by Wilson et al. [13] and Keller et al. [7].

Increased afterload shifts the curve down and to the right, which indicates that the subjects were able to maintain perfusion of tissues in spite of a significantly lower rSO_2_ that was seen during the COLD trials.

Durand et al. [6] found similar results to this investigation. A significant increase was reported in cumulative stress index which showed improved tolerance to LBNP. Researchers examined different negative pressures for 5 minutes as opposed to this investigation which used one pressure for 20 minutes. The longer exposure time showed the physiological pattern for each variable as a response to LBNP.

Cui et al. [5] examined central venous pressure with lower body negative pressure. Five-minute intervals of LBNP at various pressures were investigated as opposed to one 20-minute interval for this investigation. Results were consistent with this investigation, as Cui et al. found a significant increase in central venous pressure with cold exposure.

In summary, the subjects were able to maintain blood pressure much better in cold temperatures than in ambient one. Cold may have delayed and/or minimized the effects of lower body negative pressure on cardiovascular and thermoregulatory variables. This is in agreement with other studies that used a decrease in skin temperature as its only stressor.

## 5. Limitations

One limitation of this investigation was that a wide range of subject morphologies was observed. The weight range of our subjects was from 55.8 kg to 100.24 kg. Subjects with a similar height and weight may show trends of cardiovascular and thermoregulatory variables. This would be due to similar total fluid volume for each subject. There was also a wide range of cold tolerance noted. One possible way to address this issue would have been to pretest each individual and determine their tolerance of LBNP prior to the trials. Another possible limitation of this investigation was that oxygen saturation in the periphery was not monitored and compared to brain oxygen saturation. The significant main effect for condition may have been due to vessel constriction secondary to the cold response or it may have been due to the inability of the NIRS unit to read the true values due to vessel constriction.

## 6. Conclusions

This investigation found that cold altered the physiological response of the body to LBNP. This may be an indication that cold may delay and/or minimize the onset of shock by maintaining blood pressure for a longer period. This may provide more time for an individual to receive more advanced interventions. Further investigation is warranted to provide further support for this.

## Figures and Tables

**Table 1:** Participant characteristics (M ± SD).

Variable	M ± SD (*N* = 11)
Age (yr)	20.3 ± 2.7
Height (cm)	180.8 ± 5.7
Mass (kg)	81.6 ± 13.4
*A*_*D*_ (m^2^)	2.0 ± 0.2
% BF (skinfolds)	8.1 ± 2.3
VO_2__max⁡_ (mL·kg^−1^·min^−1^)	42.7 ± 5.1
Body density	1.1

*Note. A*_*D*_, body surface area; VO_2__max⁡_, maximal oxygen uptake.

## Abbreviations

LBNP: Lower body negative pressure
COLD: Cold trial
AMB: Ambient trial
rSO_2_: Regional oxygen saturation.
